# Complete Radiologic Response of Bulky Cerebral Metastases From Newly Diagnosed HER2-Positive Breast Cancer to Upfront Trastuzumab-Based Chemotherapy

**DOI:** 10.4021/wjon639w

**Published:** 2013-05-06

**Authors:** Daniel Brungs, Victor Sze, Louise Emmett, Richard J. Epstein

**Affiliations:** a Departments of Oncology, St Vincent’s Hospital, Sydney, Australia; b Medical Imaging, St Vincent’s Hospital, Sydney, Australia

**Keywords:** Cerebral metastasis, Blood-brain barrier, Cancer chemotherapy, Breast neoplasms

## Abstract

The blood-brain barrier is traditionally regarded as an insurmountable obstacle to the effective drug therapy of brain metastases from solid tumors. Here we describe a striking case of complete radiologic response to chemotherapy, and propose that the critical success factors include the large tumor size, HER2-positivity, and concomitant use of trastuzumab.

## Introduction

Oncology practice guidelines teach that neither cytotoxic therapy nor large-molecule target-specific therapies are routinely useful in treating cerebral metastases [1, 2] unless these arise from exquisitely chemosensitive primary tumors such as lymphomas or germ-cell neoplasms [3]. To address this unmet need, cerebral metastasectomy and/or sterotactic radiotherapy have become standards of care for most solid tumor patients with intracranial and limited extracranial metastatic disease [4], reflecting perceptions of a blood-brain barrier (BBB) that hinders transmembrane passage of drug molecules larger than 400 Da [5].

In recent years this orthodoxy has been challenged on empirical and theoretical grounds [6], with one objection being that the BBB is disrupted by intracerebral deposits larger than 1 cm [7]. HER2-overexpressing breast cancers are relevant to this debate, given that they recur preferentially within the brain [8], presumably reflecting the rich supply of HER-stimulatory heregulin ligands (neuregulins) [9] within the metastatic ‘soil’ of the central nervous system [10]. Relevant to this debate, we report here the history of a 50-year-old woman who presented with aggressive metastatic HER2+ breast cancer that required immediate upfront systemic therapy.

## Case Report

**Figure 1:**
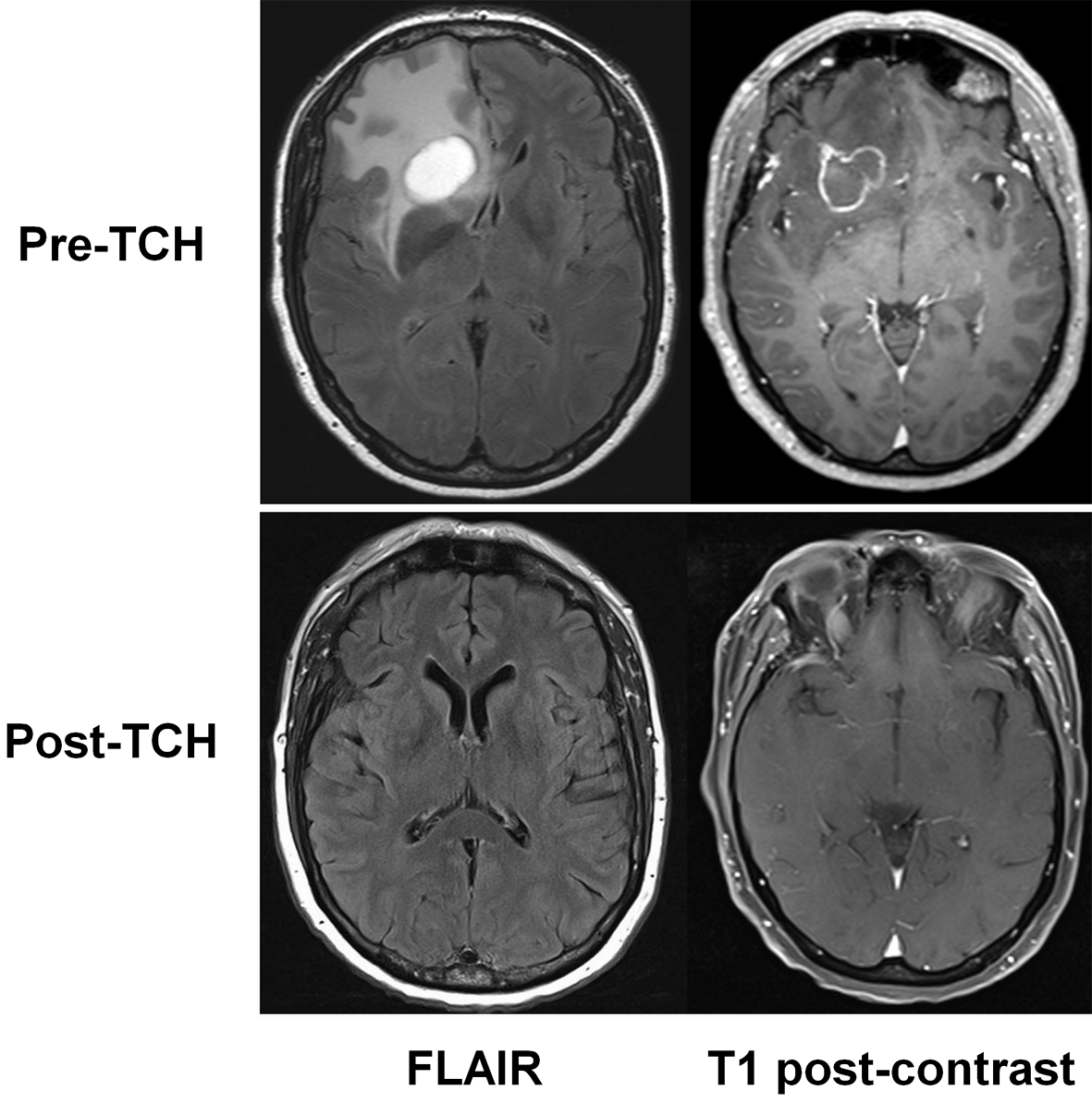
MRI appearances of brain metastases before (pre-TCH, above) and after drug treatment (post-TCH, below). Fluid attenuated inversion recovery (FLAIR) images are shown at left, and T1-weighted images at right.

A 50-year-old female presented in July 2012 after noticing thickening in the lateral aspect of the right breast. Core biopsy confirmed a 20 mm hormone receptor-negative grade 2 invasive ductal carcinoma with high levels of HER2 by both immunohistochemistry and in-situ hybridization. The patient reported no symptoms of metastatic disease, including no headaches or focal symptoms. Examination demonstrated a palpable breast mass with matted axillary lymph nodes, and normal neurological status. PET/CT scan demonstrated FDG-avid metastases in the right breast, right axillary lymph nodes, and multiple bilateral pulmonary nodules; in addition, however, a large photopenic and hypoattenuating area was detected in the right frontal lobe. CT-guided biopsy of a pulmonary nodule confirmed adenocarcinoma. Brain MRI confirmed two frontal lobe lesions (Fig. 1) (upper panels), the largest 28 mm, with extensive surrounding oedema. Gamma-knife surgery was deemed unfeasible due, but this was deemed unfeasible due to the size of the two lesions. Treatment was commenced using dexamethasone for cerebral oedema and 3-weekly standard chemotherapy using docetaxel/carboplatin plus trastuzumab (Herceptin™; DCH) with pegfilgrastim support. Repeat CT scan prior to the second cycle of DCH revealed a partial response, while a repeat MRI after five cycles showed a complete response (Fig. 1). There was also a clinical remission of the breast mass, a radiologic complete remission of the axillary adenopathy, plus near-complete remission of the pulmonary metastases. The patient continues on trastuzumab.

## Discussion

Complete responses of brain metastases to systemic therapies have been reported for kinase inhibitor therapy of lung cancer [11]; for cytotoxic chemotherapy of germ-cell tumors [12]; and in breast cancer, for hormonal therapy [13], concurrent chemoradiation plus lapatinib [14], and (for small brain secondaries only) oral capecitabine [15]. In addition, responses of leptomeningeal breast cancer have been reported using intrathecal trastuzumab [16]. Partial responses of intracerebral metastases are reported in pre-treated patients receiving tamoxifen [17], trastuzumab-radiation [18], trastuzumab-cytotoxic combinations [19], and capecitabine-lapatinib [20]. These testimonials to drug efficacy suggest that the traditional notion of the blood-brain barrier may be declining in relevance to the management of brain metastasis.

Trastuzumab reportedly exhibits poor penetration into the brain [21], and is widely believed to be ineffective in controlling brain metastases in breast cancer patients [22]. Despite this, trastuzumab induces radiosensitization in the context of cerebral metastases [23], and patients with HER2-positive brain metastases who continue trastuzumab experience longer survival [24, 25]. In contrast, the anticipated efficacy of the small-molecule HER2 kinase inhibitor lapatinib as single-agent therapy for brain metastases has proven to be marginal [26]. Indeed, based on published reports, we can infer no inverse relationship between drug molecular weight (Table 1) and clinical efficacy of brain metastasis therapy in breast cancer patients.

**Table 1:** Molecular Weights of Relevant Oncology Drugs

Drug	Reported efficacy in solid tumour cerebral metastases	MWt (Da)
Capecitabine	+	360
Carboplatin	+	371
Tamoxifen	+	372
Megestrol acetate	+	384
Gefitinib	+	447
Doxorubicin	-	580
Etoposide	+	587
Docetaxel	-	808
Lapatinib	+	944
Trastuzumab	+	148,000

The putative blood-brain barrier cut-off is 400 Da. MWt: molecular weight; Da: daltons.

In summary, just as there is growing enthusiasm for first-line use of HER2 inhibitors in lieu of chemotherapy for early breast cancer [27], so does the present case suggest that trastuzumab-based chemotherapy could come to displace surgery and/or radiation therapy in selected cases of HER2-positive bulky brain metastases. Accordingly, in this palliative context, we submit that chemonaive HER2-positive intracerebral disease should now join lymphomas and germ-cell tumors in the category of “highly chemosensitive tumors” [3].

## Abbreviations

BBB: blood-brain barrier
FLAIR: fluid-attenuated image recovery
